# 11.A. Workshop: Leveraging meso-level data to advance population health in Europe: further directions

**DOI:** 10.1093/eurpub/ckac129.687

**Published:** 2022-10-25

**Authors:** Organised by: Montpellier University Hospital (France), Chair persons: Gregoire Mercier (France)

## Abstract

Health services and public health researchers provide timely and critical evidence to answer real-world policy questions and work extensively with policymakers at the macro, meso and micro levels of government. One goal shared by researchers and policymakers is to foster evidence-informed policy and program development to ensure that policy initiatives provide the greatest benefit possible to individuals and society. Among other sources of data, meso-level datasets are usually comprising contextual data aggregated at various geographical areas such as cities, counties, and regions. Although meso-level data are sometimes used as proxies for individual level data, they can also be used to explore complex questions at the population level. This workshop aims to provide a unique, interprofessional, European conversation about how to translate meso-level research evidence into meaningful insights or recommendations. It brings together a group of high-level people from academia, think tanks, and companies who are involved in generating, transferring, or using meso-level evidence to inform public health and health care policy in Germany and France. In the first presentation, Schüttig et al. use district-level data from Germany to suggest that increased spending and improved continuity of care may be effective ways to reduce the rate of potentially avoidable hospitalizations among patients with type 2 diabetes. Then, Mercier et al. analyze department (district)-level data from France to quantify the impact of the population-based prevalence of diabetes and psychiatric conditions, of air pollution, of socio-economic variables, and of meteorological factors on the spread of COVID-19 during the first lockdown. Rodts et al, in a collaboration between a think tank and a small company, use a broad set of district-level variables to classify French ‘departements’ into homogeneous clusters in terms in needs and explore the discrepancies between total health care spending and needs at the population level. Finally, Mâlatre-Lansac et al. build on these studies to discuss how data can be used to inform public health and health care policy making in Europe. In addition, they suggest future directions to improve meso-level data-driven policy at the local, national, and European levels. Beyond methodological points, the discussion will address ethical issues in the use of meso-level data, and how to improve the availability of data, and the ability of local, regional, and national policymakers to use research evidence efficiently. It is designed as a regular workshop with 4 presentations (10 minutes each), ample audience interaction through Q&A after each presentation and one freehand poll in the introduction of each presentation.

**Key messages:**

• Meso-level data can be efficiently leveraged to inform health care policy.

• Further efforts need to be taken to address the information needs of policymakers.

